# Investigating the neurobehavioral symptoms of neuronopathic Hunter syndrome

**DOI:** 10.1016/j.ymgmr.2020.100566

**Published:** 2020-01-16

**Authors:** Nathan Grant

**Affiliations:** Department of Molecular and Cellular Biology, Department of Anthropology, Harvard University, Cambridge, MA, USA

**Keywords:** Mucopolysaccharidosis II, Hunter syndrome, Neuronopathic phenotype, Neurobehavioral symptoms, Pain, Siblings

Managing the neurobehavioral symptoms of Hunter syndrome (mucopolysaccharidosis II) can be particularly challenging. Most individuals with the neuronopathic phenotype have significant language impairment and are unable to verbally communicate the reasons for their behavior [1]. Accordingly, it is uncertain whether the neurobehavioral symptoms are indicative of a psychiatric disorder associated with Hunter syndrome or occur in response to underlying somatic complications such as pain [2]. Eisengart et al. provide an important description of the neurobehavioral symptoms of neuronopathic Hunter syndrome; however, some additional questions and implications must be considered [3].

Eisengart et al. report that neurobehavioral symptoms include hitting and biting. While these behaviors may be intentionally aggressive, they may also occur in non-aggressive contexts such as when attempting to communicate with others [3]. However, the authors do not describe the factors that helped them distinguish between a behavior that was intentionally aggressive over one that was an attempt to engage socially. This information is needed and may help elucidate the sources that stimulate these challenging behaviors.

If challenging behaviors occur in attempts to communicate with others, what are affected individuals trying to communicate? The authors report that caregivers worried pain was a source for behavioral outbursts [3]. This implies that pain management should be considered when treating neurobehavioral symptoms. More studies are needed to assess the association of pain and neurobehavioral symptoms in Hunter syndrome.

Furthermore, siblings often provide care for their brothers and sisters with mucopolysaccharidosis [4]. Eisengart et al. describe the impact of neurobehavioral symptoms on parents but do not comment on the experiences of siblings. Future studies must assess the perspectives of siblings.

While Eisengart et al. provide much-needed information, these recommendations may guide future studies to dig even deeper to understand the contributing pathophysiological process and overall impact of the neurobehavioral symptoms of Hunter syndrome.

## Funding

No funding was received.

## Declaration of Competing Interest

There are no conflicts of interest.
